# Risky decision making and cognitive flexibility among online sports bettors in Nigeria

**DOI:** 10.1002/ijop.12663

**Published:** 2020-02-03

**Authors:** Tochukwu Nweze, Ethelbert Agu, Florian Lange

**Affiliations:** ^1^ Department of Psychology University of Nigeria Nsukka Nigeria; ^2^ Behavioral Engineering Group KU Leuven Leuven Belgium; ^3^ MRC Cognition and Brain Sciences Unit University of Cambridge Cambridge United Kingdom

**Keywords:** Risky decision making, Gamblers, Impulsivity, Iowa gambling task, Cognitive flexibility

## Abstract

Online sports betting is a popular recreational activity in Nigeria. Like other forms of gambling, risk of pathological progression exists for gamblers who continue betting despite severe financial and psychosocial consequences. In the present study, we examined whether this population of gamblers shows deficits in decision making and cognitive flexibility that have been documented in Western gambling populations. Thirty‐six online sports bettors and 42 non‐gambling participants completed a version of the Iowa gambling task (IGT) and an established set‐shifting task for the assessment of cognitive flexibility. The two groups did not differ significantly in the selection of disadvantageous decks on the IGT. In contrast, sports bettors committed significantly more errors on the set‐shifting task than non‐gambling control participants. As this performance deficit was not specific to trials requiring a set shift, it most likely resulted from gambling‐related changes in general cognitive or motivational abilities that are required to successfully complete challenging mental tasks. While our results illustrate that findings from Western populations cannot automatically be generalised to other contexts, it should be noted that we focused on only one particular type of gambling and included mostly participants with mild gambling‐related problems.

Gambling is an ubiquitous activity among adolescents and young adults across the globe (Vigna‐Taglianti et al., 2017). Gambling activities can become problematic when being continued despite serious adverse economic and psychosocial consequences (e.g., Armstrong & Carroll, 2017). Identifying risk factors for and mechanisms underlying problematic gambling behaviour is important to anticipate and mitigate these consequences. One line of research on the determinants of problem gambling focuses on impairments in cognitive processes on the part of the gambler. Such impairments are often examined by means of laboratory decision‐making tasks and tests of cognitive flexibility.

A popular paradigm for the assessment of decision making under uncertainty is the Iowa gambling task (IGT; Bechara, Damasio, Damasio, & Anderson, 1994). In the IGT, participants are allowed to sample cards from four decks, which are associated with a given reward and punishment structure. Some of the decks are advantageous, while some are disadvantageous. The selection from the disadvantageous decks is associated with a negative expected value as it offers occasional large wins but also the possibility to incur even larger losses. Selecting cards from the advantageous deck, on the other hand, relates to smaller wins and losses and a positive expected value. Performance in this task requires that participants deal with this uncertainty in the context of the reward and punishment structure, as they gradually learn over the trials which decks offer more rewards than punishment in the long term. While early studies showed that patients with damage to the ventromedial prefrontal cortex (Bechara et al., 1994) and substance use disorders (Bornovalova, Lejuez, Daughters, Zachary Rosenthal, & Lynch, 2005) are impaired in the IGT, recent evidence has also identified pathological gamblers as a population encountering problems with making advantageous choices on the task (Brevers et al., 2012; Ledgerwood et al., 2011). In the IGT, pathological gamblers have an abnormal preference for the risky choice of disadvantageous decks, which offer short term rewards but long term punishment (Bechara, 2003). The inability to decide advantageously against the background of this reward structure may relate to alterations in limbic‐striatal networks involved in the processing of rewards (Everitt & Robbins, 2005) or prefrontal cortical changes affecting the control of gambling impulses (Zelazo & Muller, 2002).

Other research on the cognitive underpinnings of problematic gambling behaviour has focused on the domain of cognitive flexibility. Cognitive flexibility refers to the ability to shift cognitive sets or strategies (Miyake et al., 2000) and has traditionally been studied with neuropsychological tests such as the Wisconsin Card Sorting Test (WCST; Heaton, Chelume, Talley, Kay, & Curtiss, 1993). Patients with gambling disorder have been reported to show more perseverative behaviour on this test (van Timmeren, Daams, van Holst, & Goudriaan, 2018), that is, they tend to stick to one course of action despite having received negative feedback. Similar patterns have been observed in patients with prefrontal lesions (Demakis, 2003) or Parkinson's disease (Lange, Brückner, Knebel, Seer, & Kopp, 2018), thus contributing to the view that cognitive flexibility relies on the integrity of the prefrontal cortex and its connections with the basal ganglia (Hazy, Frank, & O'Reilly, 2007). It is important to note, however, that the WCST is not a pure task of cognitive flexibility as it involves additional cognitive processes that complicate the interpretation of WCST performance deficits (Lange, Seer, & Kopp, 2017). Simplified set‐shifting or task‐switching paradigms (Kesiel et al., 2010) have been developed as more process‐pure measures of cognitive flexibility (see Lange et al., 2018, for an analysis of the differences between the WCST and task‐switching paradigms). Such paradigms require participants to repeat a cognitive task (e.g., classifying stimuli according to their colour) on some trials and to switch to another task (e.g., classifying stimuli according to their shape) on others. Little is known about the performance of problematic gamblers on such tasks of cognitive flexibility. The only study we are aware of did not reveal differences in task‐switching performance between pathological gamblers and healthy controls (van Timmeren, Jansen, Caan, Goudriaan, & van Holst, 2016).

An important limitation of previous studies on the cognitive correlates of problematic gambling is their almost exclusive focus on gamblers from Western populations. Cultural differences can influence the initiation and maintenance of gambling in multiple ways (Raylu & Oei, 2004), implying that findings cannot automatically be generalised from one culture to another. The present study aims to probe the generalizability of previous findings by examining gambling‐related deficits in decision‐making and cognitive flexibility in a Nigerian sample. A popular form of gambling in Nigeria is sports betting with 34% of the young population reporting to place bets on sports event regularly (NOIPolls). A particular appeal of this type of gambling is the possibility to place bets online, during an ongoing sports competition, which reduces the waiting time between a bet and the outcome (Russell, Hing, & Browne, 2019). To test whether young Nigerians who regularly engage in online sports betting have difficulties making decisions under uncertainty or shifting cognitive sets, we will compare their performance on the IGT and on an established task‐switching paradigm task to the performance of non‐gamblers.

## METHODS

### Participants

A total of 78 participants took part in the study. The sample consisted of 36 individuals who reported to regularly engage in online sports betting (referred to as gamblers in the following) and 42 control participants who reported not to do so (Table 1). Demographic and IGT data were lost for one participant in the gambling group. Data of this participant were only included in the analysis of task‐switching performance. Most gamblers were recruited from naijabet shops, the predominant online sports trading industry in Nigeria, while the remaining gamblers were recruited from University of Nigeria, Nsukka, where all the control participants were recruited as well. Participants who were below 18 years and above 35 years were excluded from the study, as well as those who did not have basic knowledge in English language. This is to ensure that all participants in both groups were able to understand the task instructions written in English. Other exclusion criteria which were orally screened were history of neurological disorders, head injuries or seizures. The study was approved by the Psychology departmental review board of University of Nigeria, Nsukka.

**Table 1:** Demographic information of healthy controls and gamblers

	Controls	Gamblers
	Mean (SD)	Mean (SD)
*N*	42	36^†^
Age	22.24 (3.97)	22.69 (3.64)
Gender: Male/Female	36/6	33/2
Education (Years)	12.29 (1.69)	12.69 (1.53)
BIS	14.43 (3.44)	13.83 (3.19)
G‐SAS	‐	16.57 (9.69)
Number of years gambled	‐	2.36 (1.28)
Age of onset	‐	20.34 (3.89)
Real life net profit (Naira)	‐	1060 (1395.15)

*N* = Number of participants; BIS = Barratt impulsiveness scale; G‐SAS = Gambling symptoms assessment scale; *SD* = standard deviation.

[^†^] Missing demographics for one participant.

### Procedures and assessment

Testing was done in a conducive room within the premises of the naijabet shops in Enugu state, Nigeria or at the University of Nigeria, Nsukka, where the healthy controls were recruited. Participants consented to take part in the study by signing a form that described the purpose of the study and the expectation from the participants. They provided demographic information, filled in two self‐report scales described below for clinical background assessment, and completed two computerised tasks presented on an 14inch laptop for the assessment of decision making and cognitive flexibility.

#### *Barratt Impulsiveness Scale (BIS)*

Impulsiveness was examined with a brief and unidimensional version of the Barratt Impulsiveness Scale (BIS; Steinberg, Sharp, Stanford, & Tharp, 2013). Example items include “*I do things without thinking*” and “*I am a careful thinker*” (reverse coded). Each of the eight items was completed on a four‐point scale (1 = rarely/never; 2 = occasionally; 3 = often; 4 = almost always/always). Internal consistency of the scale was lower (α = 0.54) in the present study than in the original report by Steinberg and colleagues (α = 0.78). BIS results should thus be interpreted with caution.

#### *The Gambling Symptom Assessment Scale (G‐SAS)*

Participants in the gambling group completed the Gambling Symptom Assessment Scale (G‐SAS; Kim, Grant, Potenza, Blanco, & Hollander, 2009), a 12‐item questionnaire that measures gambling‐symptom severity during the past 7 days. Participants reported gambling‐related urges, thoughts, feelings and behaviours on a 0–4 scaling format. Maximum possible score was 48 points with the following individual score categorization: extreme = 41–48; severe = 40–31; moderate = 30–21; mild = 8–20. Kim and colleagues reported that the internal consistency of the G‐SAS is α = 0.89 (current study: α = 0.93).

#### *Iowa gambling task*

A computerised version of the IGT (Mueller & Piper, 2014) was used to assess risky decision making. Participants were presented with four similar decks that were differentially labelled A, B, C and D. They received standardised instructions informing them to choose a deck at every trial and to maximise their overall score. Choosing a deck resulted in a probabilistically determined number of points added to or subtracted from the participant's score. Two of the decks (A, B) were disadvantageous, in that they offered immediate gains but occasional huge losses resulting in an overall negative expected value. The other two decks (C, D) were advantageous because they provided small gains and small losses, leading to an overall positive outcome. Participants were informed to earn as much money as possible while freely switching between decks. The total number of trials was 100, divided into 5 blocks of 20 trials each. Performance was measured by the number of risky decks chosen at each block. Participants' overall task score served as a secondary outcome.

#### *The colour‐shape‐shifting task*

The colour‐shape shifting task has frequently been used to assess cognitive set‐shifting (Friedman et al., 2016). In designing the task for the current study (using OpenSesame version 3.1.4; Mathôt, Schreij, & Theeuwes, 2012), we closely followed the description provided by Ito et al. (2015). On the task, participants were required to classify stimuli presented on a computer screen according to one of two rules: colour or shape. Target stimuli were circles or triangles in either red or green colour. Target stimuli were preceded by a cue (presented above the target position) indicating the sorting rule to be applied on the current trial (“C” for colour, “S” for shape). Cue display started 350 ms before target display and ended with participants' keyboard response. Participants received a negative feedback sound (200 ms, 440 Hz) after incorrect responses.

The task started with two single task blocks, each consisting of 24 trials preceded by 12 practice trials and 2 warm‐up trials (excluded from analyses). In the first single‐task block, participants had to apply the colour rule: They were instructed to press “z” with the left index finger whenever a red stimulus appeared but to press “/” whenever a green stimulus appeared. In the second single‐task block, participants had to apply the shape rule: they were instructed to press “z” whenever a circle was presented and to press “/” whenever a triangle appeared. Participants then practiced switching between the two rules (24 trials) before completing two mixed‐task blocks (56 trials plus 4 warm‐up trials each). The sequence of trials within these mixed‐task blocks was pseudorandomised to ensure that (a) the colour rule and the shape rule had to be applied equally often and (b) switch trials (trials with a cue that differs from the cue of the previous trial) and repeat trials (trials with a cue that matches the cue from the previous trial) occurred with equal frequency. Error rates and mean response times were analysed separately for switch trials and repeat trials in mixed blocks as well as for repeat trials in single‐task blocks. For the calculation of response times, we excluded responses on or following incorrect trials, responses faster than 200 ms, and response times more than three standard deviations above a participant's mean response time.

### Statistical analyses

Mixed‐factor analyses of variance (ANOVAs) with Greenhouse–Geisser correction were used to examine potential group differences in performance on the IGT and the colour‐shape‐shifting task. In addition, correlation analyses were conducted to explore potential relationships between performance measures and clinical variables (e.g., BIS and G‐SAS) in our sample of gambling individuals. The level of significance was set to α = .05.

## RESULTS

Demographic and clinical data are presented in Table 1. Independent *t* tests did not show any significant group differences in age: *t*(75) = −0.51, *p* = .61; impulsiveness; *t*(75) = 0.79, *p* = .43 and education, which was measured by the number of years completed in schools; *t*(75) = −1.08, *p* = .28. The groups did not differ significantly with regard to gender distribution either, *χ* ^2^(1,75) = 1.51, *p* = .22. Most participants in the gambling group reported gambling‐related behavioural problems in the mild (*n* = 23) or moderate range (*n* = 11), with only one participant each falling into the extreme and severe symptom category on the G‐SAS. Fourteen gamblers reported net losses from gambling in everyday life (100 to 5000 Naira per week) while 21 gamblers reported net profits (100 to 10,000 Naira per week).

### Performance on the IGT

Risky decision making was examined by submitting the number of disadvantageous decks chosen on the IGT to a 2 × 5 mixed ANOVA involving the within‐subject factor Block (1st to 5th) and the between‐subjects factor Group (gamblers vs. controls). The ANOVA revealed a significant main effect of Block, *F*(2.98, 223.44) = 3.88, *p* = .01, η*p* ^2^ = .049, indicating that the number of chosen disadvantageous decks decreased from early to late blocks on the IGT (see Table 2). However, neither the main effect of Group, *F*(1, 75) = 0.21, *p* = .64, η*p* ^2^ = .003, nor the Group × Block interaction were statistically significant, *F*(2.98, 223.44) = 1.32, *p* = .27, η*p* ^2^ = .017. In a similar vein, net outcome on the IGT did not differ significantly between gamblers and controls, *t*(75) = 0.458, *p* = .65. Hence, our analyses did not reveal any substantial group differences on the IGT.

**Table 2:** Comparison of healthy controls and gamblers on IGT characteristics

	Controls	Gamblers
	Mean (SD)	Mean (SD)
Block 1	11.24 (2.31)	11.34 (2.79)
Block 2	10.17 (2.70)	11.26 (3.18)
Block 3	10.62 (3.53)	9.89 (3.84)
Block 4	9.67 (3.71)	9.74 (4.07)
Block 5	9.67 (4.49)	10.49 (4.33)
Total risky decks	51.36 (12.18)	52.20 (13.00)
Net outcome	−271.43 (634.34)	−336.43 (603.91)

IGT = Iowa gambling task; *SD* = standard deviation.

### Set‐shifting performance

Set‐shifting was examined by submitting error rates and mean response times on the colour‐shape shifting task to separate 2 × 3 mixed ANOVAs involving the within‐subject factor Trial Type (mixed switch, mixed repeat, single repeat) and the between‐subjects factor Group (gamblers vs. controls). Participants were only included in these analyses if they performed significantly better than chance (as indicated by a binomial test) on each of the three trial types. Application of this criterion led to the exclusion of 17 participants (10 controls and 7 gamblers).

**Figure 1:**
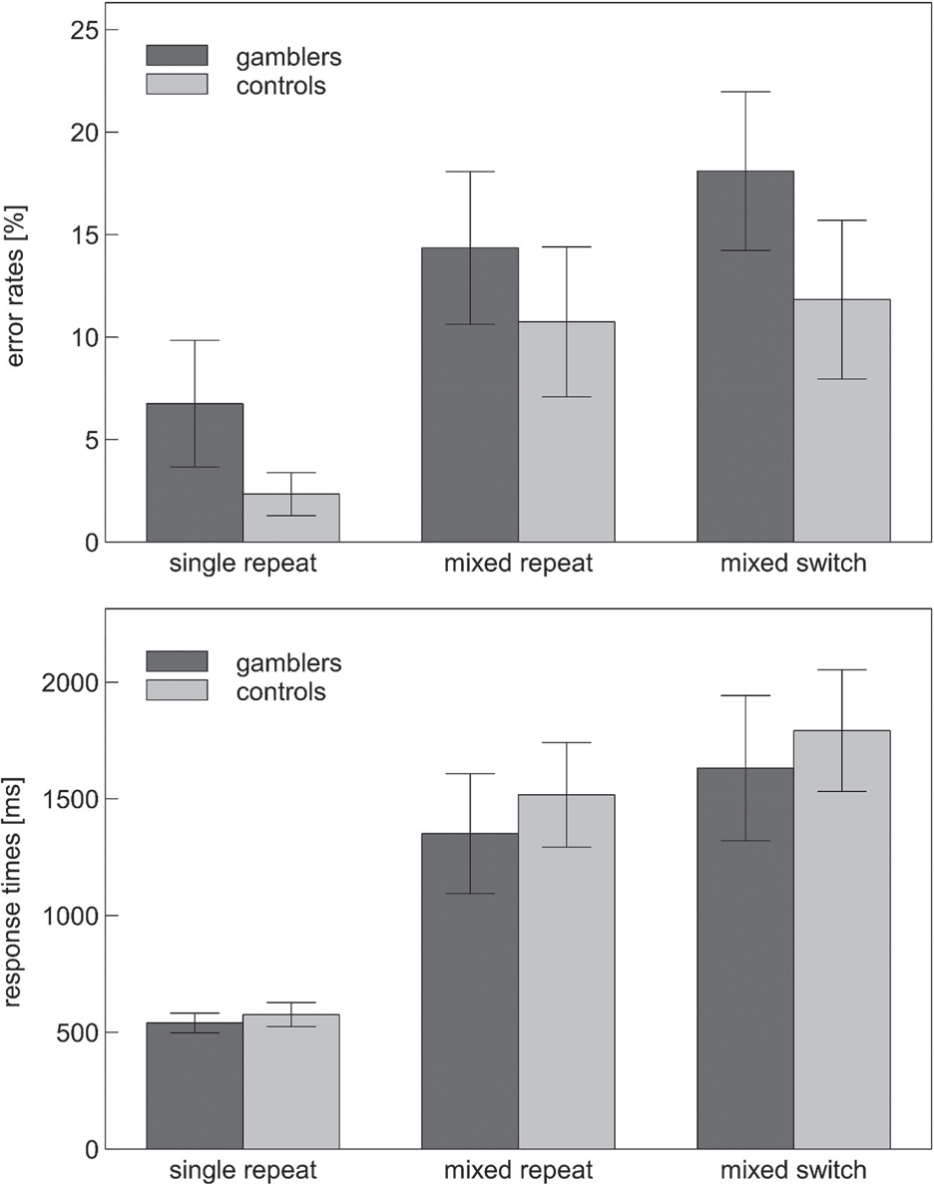
Accuracy and latency of participants' responses to repeat trials in single‐task blocks (single repeat), repeat trials in mixed‐task blocks (mixed repeat) and switch trials in mixed‐task blocks (mixed switch) of the set‐shifting task. Error bars indicate 95% confidence intervals.

The 2 × 3 ANOVA on error rates revealed a significant effect of Trial Type, *F*(1.52, 89.57) = 35.79, *p* < .001, η*p* ^2^ = .378, with error rates increasing from repeat trials in single‐task blocks (4%) to repeat trials in mixed‐task blocks (12%) to switch trials in mixed‐task blocks (15%; see Figure 1). More importantly, error rates differed significantly between groups *F*(1, 59) = 6.26, *p* = .02, η*p* ^2^ = .096, with gamblers (13%) committing more errors than controls (8%) on average. This group difference was not moderated by Trial Type, *F*(1.52, 89.57) = 0.56, *p* = .53, η*p* ^2^ = .009. With regard to response times, only the main effect of Trial Type, *F*(1.24, 73.11) = 116.83, *p* < .001, η*p* ^2^ = .66, but neither the main effect of Groups, *F*(1, 59) = 0.90, *p* = .35, η*p* ^2^ = .015, nor the Trial Type × Groups interaction, *F*(1.24, 73.11) = 0.44, *p* = .55, η*p* ^2^ = .01, were statistically significant. Hence, task performance was less accurate in gamblers than in controls, but this alteration was not specific to a particular trial type and it did not extend to the level of response times.

### Correlational analyses

Pearson correlation analyses (Tables 3 & 4) showed that IGT performance measures (i.e., net profit and the number of selected disadvantageous decks) were strongly correlated to each other, as were error measures in the mixed blocks of the set‐shifting task. We did not observe any substantial correlations between performance measures and clinical variables. The correlation between G‐SAS scores and the number of errors gamblers committed on switch trials of the set‐shifting task approached statistical significance (*r* = −.337, *p* = .08), but this correlation seems to be attributable to age being positively related to switching errors (*r* = .465, *p* = .01) and negatively related to G‐SAS scores (*r* = −.379, *p* = .05) in our sample.

**Table 3:** Pearson correlations between clinical variables, IGT performance and set‐shifting measures in the group of gamblers

Variables	1	2	3	4	5	6	7	8	9
1. BIS	‐								
2. G‐SAS	.041	‐							
3. History	−.021	.208	‐						
4. Net profit real life	.011	−.096	−.029	‐					
5. Net profit IGT	−.041	−.042	−.201	.093	‐				
6. Total risky decks	−.087	−.055	.119	−.099	−.431^†^	‐			
7. Error mixed switch	−.005	−.337	.251	.008	.109	.241	‐		
8. Error mixed repeat	−.211	−.069	.341	−.035	.197	.259	.766^†^	‐	
9. Error single	−.106	−.086	−.314	.314	.292	−.185	.050	.255	‐

*Note*: Correlations between set‐shifting measures and other variables: *n* = 28, correlations between set‐shifting measures: *n* = 29, all other correlations: *n* = 35. BIS = Barratt impulsiveness scale; G‐SAS = Gambling symptoms assessment scale; History = history of gambling measured in years; IGT = Iowa gambling tasks.

[^†^] Correlation is significant at .05 level (2‐tailed).

**Table 4:** Pearson correlations between impulsiveness, IGT performance, and set‐shifting measures in the healthy control group

Variables	1	2	3	4	5	6
1. BIS	‐					
2. Net profit IGT	−.257	‐				
3. Total risky decks	.141	−.542^†^	‐			
4. Error mixed switch	−.018	.068	.053	‐		
5. Error mixed repeat	.176	−.096	.052	.823^†^	‐	
6. Error single	.175	−.141	.293	.335	.343	‐

*Note*: Correlation between set‐shifting measures and other variables: *n* = 32, other correlations: *n* = 42; BIS = Barratt impulsiveness scale; IGT = Iowa gambling task.

[^†^] Correlation is significant at .05 level (2‐tailed).

## DISCUSSION

This study compared gamblers (online sports bettors) and non‐gamblers with regard to their performance on tasks of decision making under risk and cognitive flexibility. Contrary to findings from previous studies (Brevers et al., 2012; Goudriaan, Oosterlaan, Debeurs, & Vandenbrink, 2006), we found no significant difference in the selection of risky decks in the IGT among the two groups. Similarly, gamblers did not differ from non‐gamblers in their ability to learn from the outcome of their choices over the course of the IGT. Gamblers committed more errors on the set‐shifting task used to assess cognitive flexibility, but this deficit was not confined to trials requiring a task switch. This pattern is compatible with previous studies that found gambling‐related deficits on complex flexibility tasks (such as the WCST; van Timmeren et al., 2018), but not on more flexibility‐specific measures (such as the performance difference between switch and repeat trials in task‐switching paradigms; van Timmeren et al., 2016).

A possible explanation for the lack of a significant difference among gamblers and non‐gamblers in the selection of risky decks on the IGT is that gambling‐related deficits are too small to be reliably detected given the size of our sample. Some previous studies involving similar sample sizes did not find statistically significant group differences either (De Wilde, Goudriaan, Sabbe, Hulstijn, & Dom, 2013; Linnet, Møller, Peterson, Gjedde, & Doudet, 2010), indicating that gambling‐related IGT alterations might be smaller than often assumed and that larger samples would be necessary for a reliable analysis of these alterations. In addition, the severity of gambling might have been too mild in our sample in order to produce gambling‐related performance deficits in cognitive tasks. Compared to previous studies from western samples that have examined severe to extreme gamblers (Brevers et al., 2012), the mild gambling characteristics of our participants as indicated in their scores in gambling assessment symptoms and gambling time may explain the variance between our results and literature reported in western samples. While not being able to exclude this possibility, we did not observe IGT performance to become worse in gamblers with high symptom scores on the G‐SAS. Nonetheless, future studies could benefit from recruiting larger and more diverse samples, which would allow for informative comparisons between severe, mild and non‐gamblers. These studies should also include different types of gambling. Compared to other types of gambling (e.g., slot machine, roulette and black‐jack), decisions made during sports betting may be less similar to decisions made in the IGT. Alternatively, other decision‐making tasks that are more tailored to the contingencies involved in sports betting may be necessary to detect changes in decision making in this specific population of gamblers.

Our data suggest that the gambling population studied here has difficulties on task‐switching tasks, but that these difficulties are not specifically related to task switching. Compared to non‐gambling controls, gamblers made more errors when required to switch between two cognitive tasks, but they also made more errors when they had to complete the tasks separately. In other words, it is unlikely that the performance deficits observed on the colour‐shape‐shifting task results from a specific cognitive flexibility deficit in gamblers. Instead, they may rather be attributable to changes in general factors that are required to perform cognitive tasks at a high level of accuracy such as motivation, comprehension of task instructions, or basic cognitive abilities (e.g., stimulus categorization). Future studies are required to clarify whether similar processes can account for gambling‐related alterations in more complex tasks of cognitive flexibility (van Timmeren et al., 2018) as well.

Sports betting is a relatively new form of gambling in Nigeria, and until now, has been lawful and seen as a recreational activity. In comparison to more established forms of gambling activities (e.g., lotteries, pools and casino), sport betting may be socially more acceptable and thus attract a different type of gambler. Thus, the conclusions of this study cannot be extended to all varieties of gambling. In this context, it should also be noted that we did not assess the possibility that our betting participants also engaged in other types of gambling activities. The reason for this was that aside betting, all other forms of gambling activities are not only unpopular but highly restricted and almost entirely illegal in Nigeria. Future studies are needed to compare different forms and severities of gambling to further examine the generality of cognitive changes related to gambling and gambling problems. Such studies are also likely to benefit from the use of larger test batteries and samples and ideally, they would employ longitudinal designs to assess whether cognitive alterations predict gambling‐related problems or vice versa.

In conclusion, the present results do not support the existence of gambling‐related deficits in decision making or cognitive flexibility in Nigerian sports bettors. While there are many possibilities to account for these results, they suggest that findings on the cognitive underpinnings of gambling cannot automatically be generalised from Western populations to this specific gambling context. As such, they highlight the need for systematic cross‐cultural research efforts in studying gambling and gambling‐related problems (Raylu & Oei, 2004).

All procedures performed in this were in accordance with the ethical standards of the institutional and national research committee and with the 1964 Helsinki declaration and its amendments or comparable ethical standards. Informed consent was obtained from all individual participants included in the study.
